# Supportive medication in cancer during pregnancy

**DOI:** 10.1186/s12884-020-03432-7

**Published:** 2020-12-01

**Authors:** Flora Zagouri, Nikolaos Dedes, Alkistis Papatheodoridi, Michael Liontos, Meletios Athanasios Dimopoulos

**Affiliations:** Haematology - Oncology Unit, Department of Clinical Therapeutics, Alexandra Hospital, National and Kapodistrian University of Athens, Vasilissis Sofias 80, 11528 Athens, Greece

**Keywords:** Ooncology patients, Pregnancy supportive care, Ggranulocyte colony-stimulating factors, Erythropoiesis-stimulating agents, bisphosphonates, Aanticoagulation agents, Antiemetics, Glucocorticoids

## Abstract

While pregnancy-related malignancies are quite rare, their incidence is increasing and thus affecting more and more women nowadays. Their management, however, with both chemotherapy and supportive agents remains quite challenging and it seems crucial to define the optimal treatment for this special population. Concerning supportive medication, it is clinically significant to determine whether commonly used agents, including Granulocyte Colony-Stimulating Factors, Erythropoiesis-stimulating agents, Bisphosphonates, Anticoagulation agents, Antiemetics and Glucocorticoids are indeed effective in ameliorating chemotherapy side effects. Meanwhile, it is of great importance that the administration of any of these agents is safe for both mother and fetus. This review aims to provide a précis of the current literature regarding both safety and efficacy of all categories of supportive medication during pregnancy.

## Introduction

Pregnancy complicated by cancer is considered a rare phenomenon, however, mostly due to modern lifestyle, it is becoming ever more frequent and the challenge of managing such cases, ever more daunting[1–4]. The malignancies affecting pregnant women do not differ from those affecting age-matched non-pregnant women and are -in order of decreasing incidence- melanoma, breast cancer, cervical cancer, lymphomas and leukemias [4]. Unfortunately though, it is not easy to obtain a reliable epidemiologic profile of this situation as obstetrical and oncological registries are not uniform internationally and data concerning miscarriages and voluntary or therapeutic abortions are often missing [2]. One further limitation is that findings across studies are difficult to be interpreted and compared, as outcomes can be referring to either pregnancies or live births [2]. Nonetheless, cancer during pregnancy is believed to occur once per 1,000 pregnancies annually [4].

Regardless of the epidemiologic figures, what is the sole concern of the pregnant patient, is treatment and its possible fallout. Such therapeutic challenges call for an interdisciplinary approach in order for the most suitable strategy to be developed. In this spirit, the aspiration of this review is to revisit supportive treatment options and to investigate individual agents as to their efficacy in alleviating chemotherapy side effects, while highlighting any associated risks of their administration for either mother or fetus.

### Granulocyte Colony-Stimulating Factor

Granulocyte Colony-Stimulating Factor (GCSF) is an indispensable weapon in the therapeutic arsenal against cancer, however, some concerns with regard to its safety during pregnancy have been arisen. Most of the criticism stems from the fact that GCSF can traverse the placenta and that in animal models, complications such as miscarriage, low birth weight (LBW), and various developmental problems have been observed [5, 6]. Based on the latter, the Food and Drug Administration (FDA) has classified GCSF as “Pregnancy Category C” [6]. Nonetheless, a number of studies, mostly extracting data from the Severe Chronic Neutropenia International Registry (SCNIR), provide reassuring evidence for GCSF administration during pregnancy. Zeilder et al. mentioned that 16 pregnant women with chronic neutropenia of various etiology received GCSF for long periods and in one case through the entire pregnancy [7]. Another related study compared pregnant women with chronic neutropenia who received GCSF during pregnancy with those who did not [8]. There were no significant differences in spontaneous abortion and preterm delivery, whereas adverse events in the neonates were similar in both groups [8]. Similar conclusions were drawn from studies on pregnant women with breast cancer or lymphoma and other malignancies [9–11]. In an attempt to validate the beneficial role of GCSF for the treatment of unexplained recurrent miscarriage proposed by a previous study, a randomized, double-blind, placebo controlled clinical trial involving 150 women with a history of unexplained recurrent pregnancy loss was conducted. Although no significant increase in clinical pregnancy or live births with the use of GCSF in the first trimester of pregnancy was noted, differences with regard to miscarriages, live birth, adverse events, stillbirth, LBW, changes in clinical laboratory variables following drug exposure, major congenital anomalies and preterm births did not differ significantly between the GCSF-treated group and the placebo-treated [12, 13].

Considering all these data, GCSF is being gradually regarded as a safe therapeutic option during pregnancy, but there are no guidelines or at least a “general rule of thumb” as to when it should be prescribed. Since myelosuppressive chemotherapy can lead to profound and sometimes prolonged neutropenia, which may result in hospitalization so as to treat a bout of fever or a potentially life-threatening infection, the use of GCSF and similar agents have a main role in the primary and secondary prevention setting [14]. Neutropenia severity and specific chemotherapy regimen and their intensity are recognized risk factors for Febrile Neutropenia (FN). Thus, regimens can be classified as high, intermediate and low risk for FN. Moreover, other patient factors, such as age, performance status, comorbidities and disease burden should also be taken into account, when estimating the chance of FN. If the chance is found to be high (> 20%), then European and American guidelines alike suggest prophylactic use of GCSF during all cycles of chemotherapy to reduce the need for hospitalization for antibiotic therapy. Patients at low risk (< 10%) may also develop FN after a cycle of chemo and it is possible that a similar episode will occur in a future cycle. Therefore, GCSF can be administered as a secondary preventive measure to reduce the chance of recurrence [14]. This set of guidelines and recommendations is reasonably accurate, but perhaps it would be unwise to generalize them so as to include the pregnant population. It could be argued that physicians should be more lenient when prescribing GCSF to these patients, because of the particular list of pathogens that affect this group and the potentially devastating effects of a peripartum infection [11]. Consequently, more research is needed so as to develop a sufficient preventive strategy.

### Erythropoiesis-Stimulating Agents

Anemia is a usual finding in cancer patients, which can be attributed to the disease itself or constitute a drug related toxicity. Regardless of its origin, treatment is often mandatory, especially during pregnancy, as it can result in serious complications such as miscarriage or stillbirth, preterm delivery, placental abnormalities and fetal growth restriction (FGR), premature rupture of membranes and increased susceptibility to infection [15]. There is also evidence that gestational anemia is associated with congenital defects and lower cognitive and motor development in one-year-old children [15].

Anemia during pregnancy is usually treated with a combination of iron, folate and Vitamin B-12. In severe cases of iron deficiency anemia and only when these occur in the 2nd or 3rd trimester, intravenous solutions of iron, like ferric carboxymaltose can be used. Physicians should be cautious though, as one such agent, namely ferric gluconate preparations contain benzyl alcohol as a preservative, which is potentially harmful for the fetus [16, 17].

Other drugs like Erythropoiesis-Stimulating Agents (ESAs) are rarely required· however, this is not the case in cancer patients. Despite the need for such agents, this treatment is often withheld, as evidence regarding its safety during cancer complicated pregnancy is scarce. Epoetin alfa and Darbepoetin alfa are the two most commonly used ESAs and both are classified as “Pregnancy Category C” by the FDA, as adverse fetal outcomes have occurred in murine models receiving high doses of these agents [6, 18]. Caution should be exercised, as multi-dose vials of Epoetin alfa contain benzyl-alcohol as preservative, which is associated with the “gasping syndrome” (central nervous system depression, metabolic acidosis, and gasping respirations) [18]. Exposure to benzyl-alcohol can be in utero or mediated through breastfeeding [18]. Nonetheless, erythropoietin itself and its analogs are large molecules and are considered unable to cross the placenta and thus do not affect the fetus [15].

Literature on the profile of ESAs on pregnant cancer patients is wanting, but some conclusions concerning their safety and effects can be drawn from pregnant renal transplant recipients treated with these drugs. Although the relative body of evidence is not extensive, it is believed that ESAs are a safe therapeutic option, at least from the 14th week on, as its effects during the first trimester are unknown [19, 20]. Adverse effects such as thromboembolic events and hypertension are rare and generally manageable with suitable treatment adjustments [15]. Moreover, fetal hematologic variables and growth until the end of the 4th week seem to be within the normal range [21]. However, due to insufficient data, the Royal College of Obstetricians and Gynaecologists suggests that erythropoietin should be reserved only for women with end-stage renal disease and hemoglobin levels below 9 g/dL after 3 weeks of oral iron administration [15].

Taking all these into consideration, ESAs during pregnancy appear relatively complication free, unfortunately though, some issues arise when a malignancy is added to the equation. Erythropoietin is a recognized growth factor for malignant cells and is associated with decreased survival in patients with breast and cervical cancer, although, this increase in mortality could be partially attributed to increased occurrence of thromboembolic events [22, 23]. Therefore, it is suggested that patients undergoing myelosuppressive chemotherapy for a potentially curable cancer should be counseled as to the risks and benefits of ESAs and alternatives such as RBC transfusion or chemotherapy dose reduction be considered [6, 18]. Last but not least, there is a variety of factors, which may cause a patient not to respond to ESA treatment (extensive bone marrow involvement, a serum erythropoietin level > 500 IU/L, anemia of chronic disease, pure red cell aplasia due to anti-erythropoietin antibodies) [24–26].

### Bisphosphonates

Bisphosphonates are an important therapeutic agent against Cancer-Treatment-Induced-Bone-Loss (CTIBL), which primarily affects premenopausal women with breast cancer [27]. CTIBL is particularly important in young women, considering their long life expectancy and is more rapid and severe than bone loss associated with menopause [27]. CTIBL is mainly caused by chemotherapy with resultant ovarian failure or endocrine treatment with gonadotropin-releasing hormone [GnRH] analogs, aromatase inhibitors and tamoxifen [28]. In contrast to postmenopausal women, tamoxifen is associated with a decreased Bone Mass Density (BMD) in premenopausal women when the menstrual cycles resume after treatment discontinuation [28, 29]. In addition to that, GnRH analogs and chemotherapy-induced ovarian dysfunction have been linked to substantial bone mass loss annually [28]. Therefore, it has been suggested that some women receive antiresorptive therapy with bisphosphonates [28]. Furthermore, there is a growing body of evidence, which highlights the antineoplastic attributes of bisphosphonates in early breast cancer, as zoledronic acid has been correlated with improved disease-free survival in patients taking anastrozole or tamoxifen and decreased probability of bone metastases [28, 30, 31]. Despite the benefits of bisphosphonates, their pharmacological attributes and lack of pregnancy-relevant clinical evidence renders them as a controversial choice for this age group [32].

The pharmacokinetic and pharmacodynamic qualities of bisphosphonates have called into question their safety profile in women of childbearing age. These molecules are small in size and can traverse the placenta in rats. Although this is not the case in humans, their considerable incorporation into the bones and subsequent leaching into the bloodstream for years after discontinuation is undisputed [32]. Furthermore, murine models have shown that in utero exposure to bisphosphonates has been associated with skeletal malformations and fetal death due to hypocalcemia-attributed insufficient uterine contractions [32, 33]. Moreover, bone turnover remains suppressed for up to 5 years after alendronate and and 2 years after risendronate discontinuation respectively; thus suggesting that in order to avoid fetal exposure, a long period until pregnancy must ensue [33]. Nonetheless and despite being classified as “Pregnancy Category C” by the FDA, alendronate and risedronate have been approved for use in premenopausal women receiving glucocorticoids [34, 35].

Despite concern arising from experimental evidence, the little available data on bisphosphonate administration shortly before or during human pregnancy is inconclusive. A study by Ornoy et al. concluded that alendronate is not teratogenic, but exposed women are more likely to have a spontaneous abortion or to give birth at lower gestational age and to babies of significantly lower body weight; these findings though, could be confounded by the underlying disease [36]. A very recent case-control study established that women exposed to bisphosphonates had increased rates of spontaneous abortions and non-specific neonatal complications, while no significant correlation with teratogenic outcomes, fetal growth, weight and gestational age at birth was found [37]. As in the previous study, the preexisting condition could be a confounding factor [37]. In addition to these two relatively large studies, limited evidence from different research papers regarding the administration of bisphosphonates during or shortly before pregnancy indicate, that there is no association with serious adverse pregnancy outcomes [38–41]. In spite of that, additional research on this topic is needed, so as to clarify the effects of bisphosphonates on the fetus and to develop adequate guidelines addressing screening, prevention, and treatment of bone disease in pregnant women, affected by cancer or its therapies.

### Anticoagulation

Physiological alternations during pregnancy create an ideal environment for clot formation. Yet, this situation is further aggravated in the presence of cancer [42]. As pregnancy progresses, the risk is inflated [43]. Fibrinogen may have risen to 4 g/L at the end of the third trimester, with an accompanying increase of von Willebrand’s factor and of thrombogenic factors VII, VIII, IX, X and XII, whereas factors III, IV, XI, plasminogen activator inhibitor and protein S are decreased [43, 44]. Meanwhile, the gradually expanding uterus applies pressure on the pelvic veins and the inferior vena cava and thus creating flow abnormalities [45, 46]. This combination of parameters accounts for a 5-fold increase in the chance of Venous Thromboembolism (VTE) to occur by virtue of just being pregnant [44]. If pregnancy is complicated by cervical or ovarian cancer, Hodgkin’s disease or myeloid leukemia the chance of a VTE is 10 times higher than in uncomplicated pregnancies [42].

Prophylaxis and management of VTE in pregnant cancer patients constitutes a therapeutic challenge, as a variety of different factors need to be considered and a multidisciplinary approach must be taken, so as to develop a personalized treatment strategy.

First of all, the population for which prophylaxis is indicated must be outlined. All antepartum women should be surveilled for the signs and symptoms of VTE, although primary prophylaxis should be administered to a selected population considered to be at high risk for VTE [47, 48]. Primary prophylaxis for cancer patients is reserved for those who are hospitalized and perhaps those outpatients with a Khorana score ≥ 2 [49, 50]. Secondary prophylaxis is indicated for all pregnant women and cancer patients· for the former, anticoagulants should be continued for at least 6 weeks postpartum for a minimum of three months and for the latter, anticoagulation should be administered as long as the disease is considered active [47, 50]. To our knowledge though, no guidelines concerning thromboprophylaxis in pregnant cancer patients exist.

Secondly, the choice of therapeutic agents must be taken into account. Factor Xa inhibitors (e.g., rivaroxaban, apixaban, edoxaban) and oral direct thrombin inhibitors (dabigatran) are generally avoided during pregnancy, because they can cross the placenta and their effect on the fetus is unknown [51]. Furthermore, warfarin is a recognized teratogen and is designated as “Pregnancy Category X” for pregnant women except for women with mechanical heart valves, for which it is “Pregnancy Category D” [52]. Commenting on the topic of warfarin administration in pregnant women with mechanic heart valves, some researchers pointed out, that adverse fetal outcomes did not differ significantly between heparin treated women and those treated with warfarin at doses ≤ 5 mg daily [53]. The same meta-analysis highlights that warfarin was associated with a lower risk of adverse maternal outcomes than heparin [53]. Nonetheless, the established drug of choice in pregnancy and cancer is heparin, mostly Low-Molecular-Weight Heparin (LMWH) and to a lesser degree the older Unfractionated Heparin (UFH) [47, 49, 50, 54, 55]. In the rare case of Heparin Induced Thrombocytopenia or hypersensitivity reactions, alternatives to LMWH would be danaparoid, fondaparinux or argatroban [44, 47–49, 54].

Although unavailable in the United States, danaparoid is recommended by the 2012 American College of Chest Physicians guidelines as the anticoagulant of choice for women, who had severe allergic reactions to heparin, because it does not cross the placenta and rarely produces Heparin-Induced Thrombocytopenia (HIT) [47]. If danaparoid cannot be taken, fondaparinux should be administered instead [47]. In spite of the limited data on this background, it has comparable efficacy to LMWH in preventing pregnancy-related VTE, with no associated increase in birth defects, severe bleeding related complications, or serious allergic reactions [47, 56–58]. In regard to its pharmacokinetics, no placental passage of fondaparinux was demonstrated in a human cotyledon model, however in a later study, anti-Xa activity was noted in the blood of infants of mothers treated with fondaparinux [47]. In addition, most of the available data are derived from use during the second trimester or later and evidence concerning exposure during organogenesis is scarce [47]. In one such case, fondaparinux was started at the 7th week of gestation, but the patient willingly terminated the pregnancy at the 23rd week due to fetal abnormalities (Tetralogy of Fallot and Dandy– Walker syndrome) [59]. Another feasible choice in this context is argatroban, a small molecule able to cross the placenta. There are a few case reports of its use in the second and third trimester and despite the fact that no anomalies were mentioned, its safety profile is uncertain [47]. Unfortunately though, argatroban requires continuous intravenous administration and is monitored by the activated Partial Thromboplastin Time (aPTT), which must be increased by 1.5 to 3 times the baseline in order to be protective [60].

Nonetheless, when the risk of hemorrhage is very high or the anticoagulation therapy does not suffice, the placement of an Inferior Vena Cava Filter could be considered as a last resort, since data on its efficacy is questioned and its complications considerable.[47, 61].

Finally, consideration of the risk and extent of hemorrhage is crucial during labor and delivery. Women on anticoagulation going into labor are at increased risk of hemorrhage and spinal hematoma if a neuraxial anesthesia catheter is inserted [62]. To address this issue, when labor is expected, subcutaneous LMWH should be switched to intravenous UFH, which can be discontinued 4 to 6 hours before delivery, so neuraxial anesthesia can be administered once aPTT returns to normal [47, 63]. Nevertheless, arrangements for immediate blood transfusion should be made, especially in cases of sudden onset of labor and cesarean Sect. [64, 65].

### Antiemetics

Chemotherapy-Induced Nausea and Vomiting (CINV) constitute important adverse effects of treatment and the probability of its occurrence corresponds directly to the intrinsic emetogenicity of the agents used[66, 67]. Thus, in order to guide the choice of antiemesis agents or combination thereof, the various regimens are classified into high (> 90%), moderate (30–90%), low (10–30%), and minimal emetic risk (< 10%) [66, 67]. Yet, the time of onset of nausea and vomiting is also of clinical importance, because drugs may differ in their ability to prevent bouts, whether emesis is acute, delayed or anticipatory [66, 67]. Although there is a wide variety of agents with antiemetic properties, the set used in patients receiving chemotherapy is rather specific and consists of 5-HT3 receptor antagonists (ondansetron, palonosetron etc.), neurokinin (NK)-1 receptor antagonists (aprepitant, fosaprepitant etc.), glucocorticoids and olanzapine [66–70]. Dopaminergic antagonists like prochlorperazine and metoclopramide have also been used in the past, while nowadays they are reserved as rescue therapy due to their relatively limited efficacy and the adverse effects from dopaminergic blockade associated with prolonged administration [66, 71, 72].

Nausea and vomiting are quite common during the first trimester of pregnancy and despite the fact that no chemotherapy is given before the 14th week of gestation, valuable clinical evidence regarding the safety of some antiemetic agents has been gathered [73, 74]. Hyperemesis gravidarum (intractable vomiting, weight loss with volume depletion, ketonuria and/or ketonemia) affects up to 2.3% of pregnancies and is usually treated with a combination of doxylamine succinate and pyridoxine hydrochloride. Unfortunately though, these drugs have not been tested in CINV [75, 76].

Recently, ondansetron has been investigated as to its efficacy against nausea and vomiting during pregnancy, as well as its possible effects on the fetus. Ondansetron has been found to be superior to metoclopramide and the combination of doxylamine and pyridoxine in the treatment of nausea and vomiting of pregnancy and hyperemesis gravidarum [77, 78]. Furthermore, there are isolated case reports of possible association with abnormalities, like cleft palate, septal defects and renal agenesis-dysgenesis, which are not confirmed by larger studies [79–82]. Moreover, there seems to be a consensus that ondansetron is not teratogenic, while no link between in utero exposure and spontaneous abortion, stillbirth, major birth defects, preterm delivery, delivery of a LBW or small-for-gestational-age infant were found in a well-conducted Danish study [81, 83]. Nonetheless, when exposure occurs during the first trimester, the concern regarding increased incidence of oral clefts cannot be easily dismissed, as the largest study in this field did not find any correlation with any defect, except for this one [84].

Another agent which has been examined sufficiently as to its compatibility with pregnancy and its potency against nausea and vomiting is metoclopramide. In utero exposure to metoclopramide during the first trimester was not associated with significantly increased risks of major congenital malformations, LBW, pre-term delivery or perinatal death [85]. In addition, a very large study comparing pregnancy outcomes between exposed and un-exposed mothers discovered no link between exposure and 20 individual major congenital malformation categories, spontaneous abortion or stillbirth [86]. Despite unanimity regarding metoclopramide’s safety during pregnancy, there are some serious issues concerning maternal side-effects. Although comparable to ondansetron, metoclopramide is associated with increased frequency of drowsiness and dry mouth [87]. What is far more important though, is that prolonged administration -beyond 12 weeks- is correlated with the development of irreversible tardive dyskinesia (FDA black box warning), especially in females [81, 88].

In the same spirit, glucocorticoids have been studied as to their antiemetic values and their possible effect on pregnancy outcomes. Glucocorticoids have been used in the treatment of hyperemesis gravidarum with rather mixed results [87, 89]. Nonetheless, they are appropriate as monotherapy in low-emetogenicity regimens, while they appear to have a synergistic effect with 5-HT3 receptor antagonists and are thus part of the antiemetic treatment indicated for higher emetogenicity schemes [66, 67, 90–92]. Similarly to metoclopramide, they have been associated with oral cavity abnormalities, but the risk seems to be elevated only when exposure has occurred during the first trimester of gestation [93].

Finally, NK-1 receptor antagonists and olanzapine have been scrutinized as to their ability to prevent nausea and vomiting, as well as their compatibility to pregnancy. Both these agents are very effective antiemetics when used in combination with the aforementioned drugs, as they significantly decrease the chance of acute and delayed emesis, associated with highly emetogenic regimen [94–97]. Unfortunately, there is not a great amount of data regarding the safety of NK-1 receptor antagonists during pregnancy, but the 1st generation agent aprepitant has been classified as “Pregnancy Class B” by the FDA [68]. On the other hand however, olanzapine has been thoroughly studied in the context of gestation. Olanzapine has a 72% placental passage ratio, but in utero exposure even during the first trimester was not correlated to increased rates of spontaneous abortions, stillbirths, and major congenital malformations [98, 99]. Nonetheless, there are indications that exposure to second generation antipsychotics may be linked to elevated incidence of atrial and ventricular septal defects of the newborn [100]. There are also studies investigating the effect of these drugs on birth weight and psychokinetic development; more research is needed however and the available body of evidence is inconclusive [100–103].

Despite the rather reassuring profile of aprepitant and olanzapine, the present guidelines on supportive medication for gynecologic cancer in pregnancy have approved for use only 5-HT3 antagonists, metoclopramide, methylprednisolone, prednisolone or hydrocortisone and only when necessary [104].

### Glucocorticoids

After 14 weeks of gestation, administration of a number of chemotherapy drugs is feasible, but some of the most widely used, like platinum derivatives, taxanes and etoposide present considerable incidence of infusion reactions[104, 105]. In order to manage or to avoid such predicaments, glucocorticoids have been instated as an essential component of chemotherapy premedication [105].

Nonetheless, glucocorticoid pharmacokinetics and pharmakodynamics should be taken into account, as fluorinated agents like betamethasone and dexamethasone readily cross the placenta, while non-fluorinated forms like prednisolone, methylprednisolone and prednisone are converted to inactive metabolites by placental 11b-hydroxysteroid dehydrogenase, with fetal uptake being limited further by active retrograde transport through P-glycoprotein [106, 107].

Rheumatic diseases, adrenal insufficiency, asthma and prevention of neonatal respiratory distress syndrome are just a few cases in which glucocorticoids are administered to pregnant women and thus, adequate data about their behavior during pregnancy has been amassed [108–111]. However, despite the indisputable benefits that these drugs offer, there are some concerns that should be taken into consideration.

There is suspicion about the potential correlation between continuous antenatal exposure to glucocorticoids and adverse pregnancy outcomes and maternal morbidity. Evidence from experiments on rodents and an older study suggest that there is a statistically significant increase in orofacial clefts when exposure has occurred during the first trimester, but in stark contrast to them, recent findings do not confirm this link [112–115]. Moreover, data from pregnant patients with asthma on oral glucocorticoids indicate increased risk of preterm birth and low birth weight [116]. Furthermore, in utero exposure to glucocorticoids has been associated with raised risk of premature rupture of the membranes and fetal growth restriction, while on the mother’s side, the chance of pregnancy-induced hypertension, gestational diabetes, osteoporosis, and infection seems elevated [117–119]. In addition, prenatal treatment of congenital adrenal hyperplasia with dexamethasone may be linked to a negative effect on the executive functions of the offspring, but this effect is not well-established [120].

The administration of single high doses of glucocorticoids antenatally have been found to promote lung maturity, when preterm labor is a possibility (gestational age between 22 + 0 weeks and before 33 + 6 weeks) [109]. In this context, considerable research has been conducted, the findings of which are in conclusive. The most consistent finding is decreased variability of fetal heart rate on the second and third day after administration, while findings regarding umbilical blood flow are conflicting [121–123]. Moreover, while there seems to be no increased risk of neonatal infection, there are some clues that exposed neonates have a reduced stress related adrenal response; a finding of unknown importance [109, 124]. Most peculiar is the histologic observation, that there seems to be some effect on the hippocampus, as exposed neonates showed a lower density of neurons in this area, but particularly large neurons as compared with the ones of the controls [125]. Nonetheless and in spite of the relative scarcity of data, in utero exposure to high dose glucocorticoids for lung maturity seems to be free of long-term adverse effects [126, 127].

## Conclusions

After having considered all categories of supportive care agents, it is clear that their efficacy in managing chemotherapy fallout is unquestionable. However, some concerns regarding mostly fetal safety still remain. Therefore, further relevant research is needed so as expand our understanding of the behavior of these drugs in the context of pregnancy and to ultimately help develop evidence-based guidelines for this special population of cancer patients.

## Data Availability

Data sharing is not applicable to this article as no datasets were generated or analysed during the current study.

## Abbreviations

GCSF: Granulocyte Colony-Stimulating Factor
FDA: Food and drug administration
SCNIR: Severe Chronic Neutropenia International Registry
FN: Febrile neutropenia
ESAs: Erythropoiesis-stimulating agents
FGR: fetal growth restriction
CTIBL: Cancer-Treatment-Induced-Bone-Loss
VTE: Venous Thromboembolism
LMWH: Low-Molecular-Weight Heparin
UFH: Unfractionated Heparin
CINV: Chemotherapy-induced nausea and vomiting
